# Strain-Specific Epigenetic Regulation of Endogenous Retroviruses: The Role of *Trans*-Acting Modifiers

**DOI:** 10.3390/v12080810

**Published:** 2020-07-27

**Authors:** Jessica L. Elmer, Anne C. Ferguson-Smith

**Affiliations:** Department of Genetics, University of Cambridge, Cambridge CB2 3EJ, UK; jle44@cam.ac.uk

**Keywords:** ERVs, epigenetic regulation, strain-specific, modifiers, metastable epialleles, KRAB zinc finger proteins, transgenes

## Abstract

Approximately 10 percent of the mouse genome consists of endogenous retroviruses (ERVs), relics of ancient retroviral infections that are classified based on their relatedness to exogenous retroviral genera. Because of the ability of ERVs to retrotranspose, as well as their *cis*-acting regulatory potential due to functional elements located within the elements, mammalian ERVs are generally subject to epigenetic silencing by DNA methylation and repressive histone modifications. The mobilisation and expansion of ERV elements is strain-specific, leading to ERVs being highly polymorphic between inbred mouse strains, hinting at the possibility of the strain-specific regulation of ERVs. In this review, we describe the existing evidence of mouse strain-specific epigenetic control of ERVs and discuss the implications of differential ERV regulation on epigenetic inheritance models. We consider Krüppel-associated box domain (KRAB) zinc finger proteins as likely candidates for strain-specific ERV modifiers, drawing on insights gained from the study of the strain-specific behaviour of transgenes. We conclude by considering the coevolution of KRAB zinc finger proteins and actively transposing ERV elements, and highlight the importance of cross-strain studies in elucidating the mechanisms and consequences of strain-specific ERV regulation.

## 1. Introduction

Endogenous retroviruses (ERVs), a subclass of transposable element (TE), constitute approximately 10 percent of the mouse genome and arise either as a result of the successful integration of an ancient exogenous retrovirus (XRV) into the germline of the host or, more commonly, due to the retrotransposition of a previously integrated proviral sequence [1,2]. ERVs are classified based on the sequence of their reverse transcriptase gene and their relatedness to the seven XRV genera—*Gamma-* and *Epsilonretrovirus*; *Alpha-*, *Beta-*, and *Deltaretrovirus;* and *Spumaretrovirus*—into class I, II, and III ERVs, respectively [3,4,5,6]. In the mouse, class I ERVs include murine leukaemia viruses (MLVs), class II ERVs include early transposon/*Mus musculus* type D retrovirus (ETn/MusD) and intracisternal A-type particle (IAP) elements, and class III ERVs include mouse endogenous retrovirus type L (MERV-L) elements [5].

Full-length ERVs consist of 5′ and 3′ long terminal repeats (LTRs) that flank internal viral genes (*gag*, *pol*, and, in some elements, *env*) which are both essential and necessary for autonomous retrotransposition [7,8]. Non-autonomous elements, such as ETn elements, lack the reverse transcription and integrase machinery required for transposition and thus, mobilisation of these elements relies on *trans*-acting transposases encoded by other TEs. In fact, after IAPs, non-autonomous ETn elements are responsible for the second highest number of murine germline mutations of any transposon type and mobilise using the machinery of autonomous MusD elements [9,10]. Similarly, the non-autonomous IΔ1-type IAP, which has a 1.9kb deletion in *gag*-*pol*, accounts for the majority of IAP insertional mutations [11]. Whilst the majority of ERVs exist as solo LTRs which arise through inter-LTR homologous recombination, these fragmented elements still pose a significant threat to genomic integrity. This is due to the functional regulatory sequences contained within LTRs, as well as their mobilisation potential through “hijacking” the machinery of transposition-competent full-length elements [12]. In total, ERVs mainly of class I and II types are responsible for up to 12% of germline mutations in mice [11]. Given the mutagenic potential of ERVs, both full-length elements and solo LTRs are targeted for silencing via epigenetic mechanisms, as outlined in further detail below.

In inbred laboratory strains of mice, there has been a historic, and ongoing, expansion of ERV families, most notably of IAP and ETn/MusD elements, which are highly polymorphic between strains [13,14,15,16]. The susceptibility of the mouse genome to IAP mobilisation is strain-specific, with 84% of germline IAP mutations (for which a strain of origin for the TE mutation could be determined) occurring in the C3H genetic background [9]. Indeed, strain-specific diverse regions (SSDRs), which show a higher diversity between strains than is normally seen between mouse and rat and account for between 0.5% and 2.8% of the mouse genome, are enriched for recent long interspersed nuclear element (LINE) and LTR insertions [17,18]. While there is little evidence so far that strain-specific TE variants act as causal effectors of strain-specific gene expression changes or quantitative trait loci (QTL), it is perhaps notable that intronic TE variants are more frequently associated with differentially expressed genes than would be expected by chance [15].

The term “modifier gene” defines genetic variants which alter the phenotypic outcome of an independent locus, but which have no phenotypic consequence of their own [19,20]. Strain-specific morphological, physiological, and behavioural differences are well recognised, but the mechanisms underlying inter-strain variation and the causative modifier loci remain largely uncharacterised due to technical and practical limitations.

In this review, we discuss the key players in the silencing of ERVs, the existing evidence for the mouse strain-specific epigenetic control of ERVs, and the implications of differential ERV regulation on epigenetic inheritance. We reflect on lessons learned from the strain-specific behaviour of transgenes and discuss the potential mechanisms by which the strain-specific epigenetic silencing of ERVs is likely to occur.

## 2. Epigenetic Regulation of ERVs

Specific ERV classes are silenced by distinct epigenetic mechanisms in the early embryo and embryonic stem cells (ESCs). In embryonic cells, class I and II ERVs are enriched for H3K9me3, a mark deposited by the histone methyltransferase SETDB1 [21,22]. These ERVs are targeted for silencing in a sequence-dependent manner by Krüppel-associated box domain zinc-finger proteins (KRAB-ZFPs), which make up a large family of DNA binding proteins whose sequence specificity is determined through their C-terminal zinc finger arrays [23,24]. Through their KRAB domain, KRAB-ZFPs recruit KRAB-associated protein 1 (KAP1), which acts as a scaffold for other components of the transcriptional silencing machinery, including SETDB1, HP1 (heterochromatin protein 1), NuRD/HDAC (nucleosome remodelling and deacetylase complex), and DNA methyltransferases [25,26,27]. For a more detailed description of KRAB-ZFP structure and function, we refer the reader to the following comprehensive reviews [27,28]. In mouse ESCs and primordial germ cells (PGCs), whilst DNA methylation is dispensable for ERV silencing as evident from bulk ERV type analysis, knocking out SETDB1 or KAP1 results in the upregulation of several class I and II ERVs [21,22,29,30]. In contrast, DNA methylation is essential for ERV silencing in differentiated cell types and later embryonic time points, though the extent of this is not known [31,32,33]. The silencing mechanisms for class III ERVs are less clear; in ESCs, class III ERVs are largely devoid of H3K9me3 except at MERV-Ls, whose silencing and H3K9me2/3 deposition is dependent on G9a/GLP activity [34,35]. A role for the lysine-specific histone demethylase LSD1/KDM1A in MERV-L silencing in early embryos and ESCs has been proposed, but is less well defined [36,37].

In the mouse, as in other mammals, there are tightly regulated periods of epigenetic reprogramming whereby epigenetic modifications are globally erased and developmental potency is re-established. This occurs immediately post fertilisation in the zygote and in PGCs and is generally recapitulated in ESCs in vitro [38]. During development, the genome-wide removal of DNA methylation results in the transient transcriptional activation of certain classes of ERVs, notably class III MERV-Ls, which play a role in zygotic genome activation (ZGA) at the 2-cell stage in mice [39,40,41]. Retrotransposition inhibition and the transcriptional control of other classes of ERVs is maintained during epigenetic reprogramming implicating additional silencing pathways [42,43,44,45,46]. Other epigenetic mechanisms associated with ERV silencing involve 3′ tRNA fragments [47], piRNA pathways [48], and histone variants [49,50,51,52].

## 3. Evidence of Strain-Specific ERV Control

The high prevalence of polymorphic ERVs raises many questions about the similarities and differences in transposon regulation between strains. The strain-specific expansion of ERVs in inbred mice hints at the possibility of strain-specific ERV silencing, or lack thereof, and subsequent mobilisation. Thus far, no unbiased or genome-wide screens to assess strain-specific ERV control have been carried out. The few documented instances of strain-specific modifiers were identifiable due to obvious and observable phenotypic differences. These are discussed in more detail below.

### 3.1. Dactylaplasia-Causing MusD Insertions at Fbxw4

**Figure 1:**
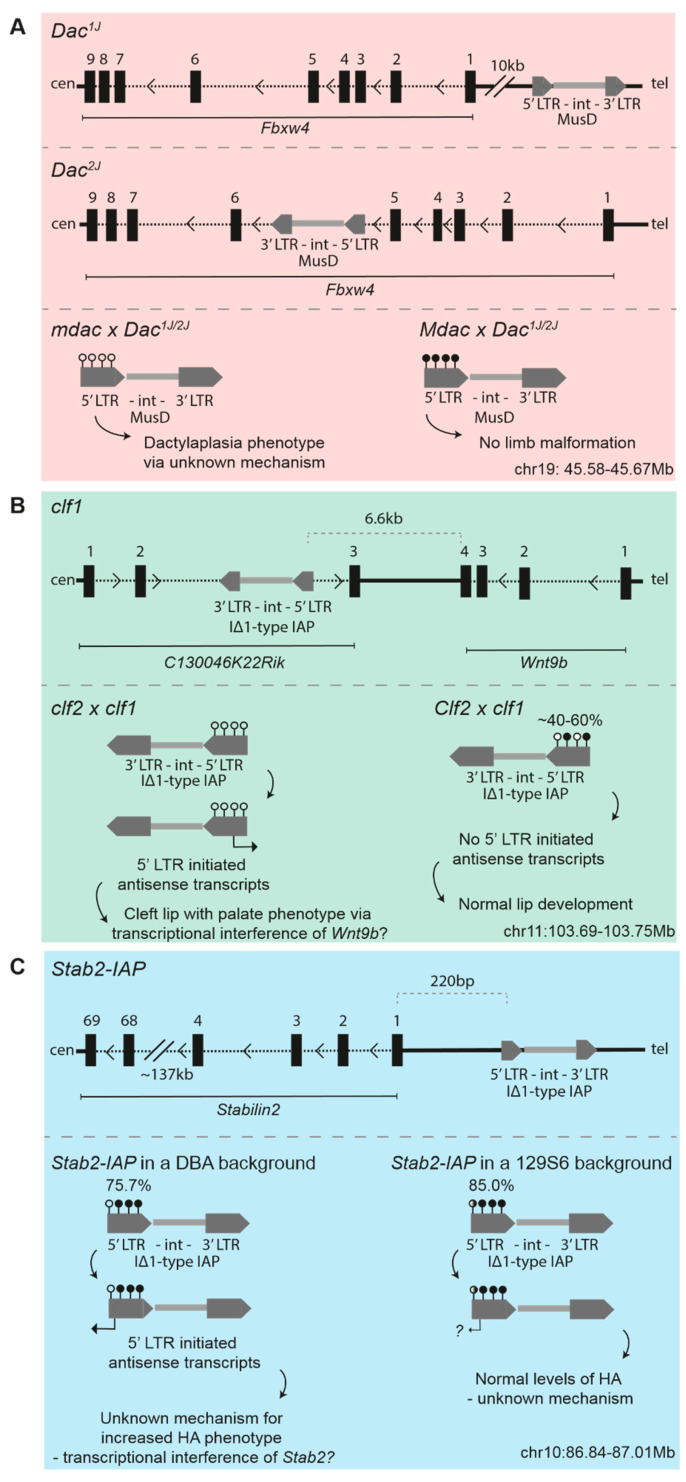
Schematics for the insertion sites of endogenous retroviruses (ERVs) subject to strain-specific regulation (upper panel) and the effects of strain-specific modifier activity (lower panel) for (**A**) *Dac^1j^* and *Dac^2j^*, (**B**) *clf1*, and (**C**) *Stab2-IAP*. Sticks with closed circles represent methylated CpGs in the long terminal repeat (LTR) of the ERV; sticks with open circles represent unmethylated CpGs. Black dotted lines depict introns; thick black lines depict intergenic regions. The information is based on the latest patch release of the 2011 mouse assembly on the UCSC Genome Browser (GRCm38.p6); the coordinates given are mm10.

Dactylaplasia is an inherited limb malformation which manifests as the absence of phalangeal bones in the middle digits of each foot in mice. The first identified mutation (*Dac^1j^*) arose in the SM7B/SC inbred strain and was found to be a homozygous lethal dominant allele [53]; a second dactylaplasia-causing mutation (*Dac^2j^*) was reported several years later on the MRL/MpJ genetic background [54]. Fine mapping and sequencing experiments established that both *Dac^1j^* and *Dac^2j^* are due to independent, full length, highly similar (99.6% identical), and polymorphic MusD element insertions, which lie either 10kb upstream (*Dac^1j^*) or within an intron (*Dac^2j^*) of the *Fbxw4* gene locus, a member of the F-box/WD40 gene family involved in protein ubiquitination and degradation [54,55,56,57] (Figure 1A, top and middle panel). The mechanism by which these MusD insertions cause dactylaplasia remains unknown [56,57].

**Figure 2:**
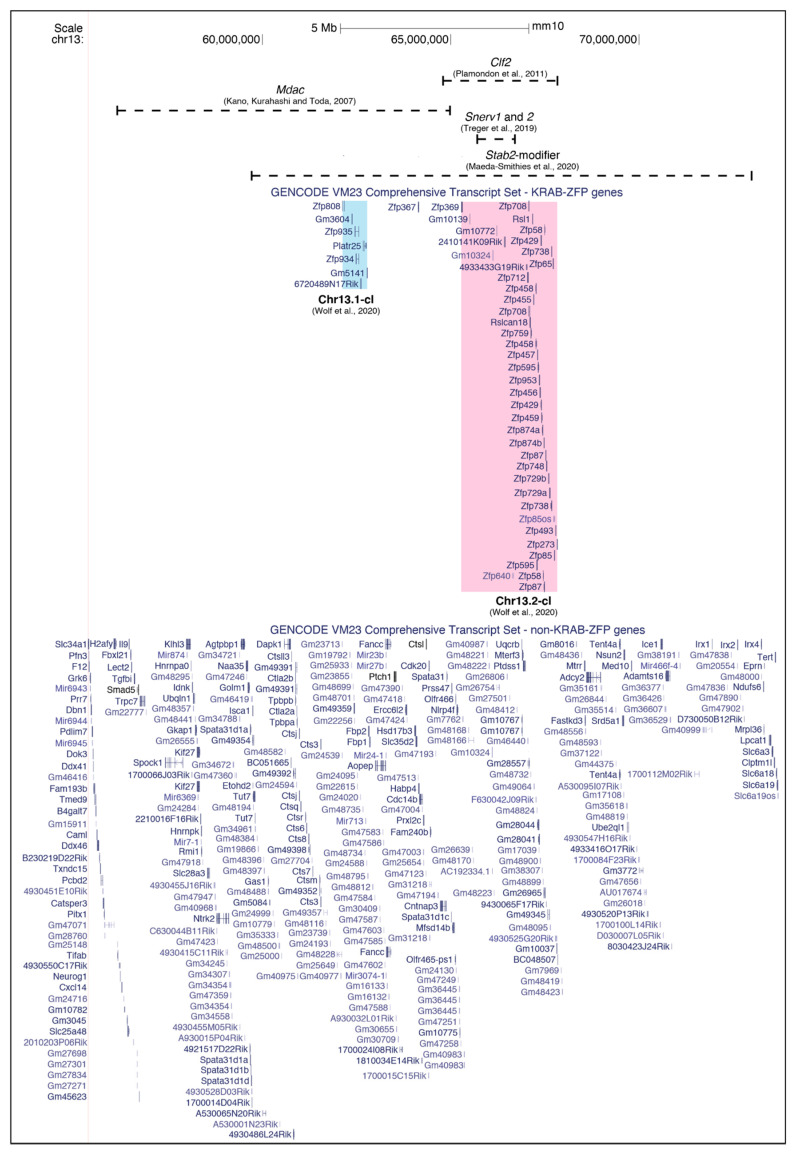
The mapped intervals of four strain-specific ERV modifiers—*Mdac*, *Clf2*, *Snerv1* and *Snerv2* and *Stab2-modifier*—on Chromosome 13. The underlying genes are separated into Krüppel-associated box domain zinc-finger protein (KRAB-ZFP) genes and non-KRAB-ZFP genes; the KRAB-ZFP clusters are highlighted and named as in [59]. Multiple isoforms are not shown.

The effects of both *Dac^1j^* and *Dac^2j^* were found to be modified by an unlinked allele *Mdac* (*modifier of Dac*), which resulted in highly polymorphic phenotypes between inbred mouse strains [53,54,55]. The strains carrying the *Mdac* allele are hypermethylated and enriched for H3K9me3 at the 5′ LTRs of *Dac^1j^* and *Dac^2j^*, resulting in the loss of aberrant MusD expression at the apical ectodermal ridge in the limb bud seen in *mdac* strains and enabling normal limb development to occur [56] (Figure 1A, bottom panel). The *Mdac* locus was first mapped to a 28Mb interval on Chromosome 13 and was later refined to a 9.4Mb region containing 125 genes, including many known to be important for limb development—e.g., *Ror2*, *Msx2*, *Fgfr4*, and *Patched* [55,56] (Figure 2). This 9.4Mb region contains a KRAB-ZFP cluster of six KRAB-ZFP genes which, given their known role in ERV epigenetic targeting, are possible *Mdac* candidates [58]. It is worth noting, however, that a recent study where this KRAB-ZFP cluster (Chr13.1-cl KO) was deleted did not lead to a global upregulation of MusD elements in ESCs [59]. This is in line with the finding that a control MusD element was not differentially regulated according to the *mdac*/*Mdac* genotype [56].

### 3.2. Cleft Lip Palate-Causing IAP Insertions at Wnt9b

Nonsyndromic cleft lip with palate (CL/P) defects arise when the medial and frontal nasal prominences fail to fuse; CL/P causes neonatal lethality in mice, as the pups are unable to suckle. CL/P spontaneously occurs with a 20–30% frequency in the inbred mouse strain A/WySn, making it a commonly used model to study clefting [60]. Similarly to the dactylaplasia phenotype, the CL/P phenotype in A/WySn mice involves two unlinked genes, a recessive mutation (*clf1*), and a second semi-dominant modifier locus (*Clf2*) [61]. The *clf1* mutation was identified as an IAP element insertion into a ncRNA, *C130046K22Rik*, located 6.6kb downstream of *Wnt9b* [62,63] (Figure 1B, upper panel). The insertion is only present in “A” strains and is a IΔ1-type IAP, the same type responsible for the *Agouti viable yellow* (*A^vy^*) and *Axin-fused* (*Axin^Fu^*) metastable epialleles (discussed below). *Wnt9b* has been implicated in clefting previously: *Wnt9b* null embryos have deficient growth of the facial prominences, resulting in CL/P, which possibly manifests via the downregulation of Fibroblast Growth Factor (FGF) signalling in these mutants [64].

In A/WySn embryos with CL/P, the 5′ LTR of the *clf1* IAP element is unmethylated [63,65,66,67] (Figure 1B, lower panel). 5′ LTR initiated antisense IAP transcripts and reduced *Wnt9b* levels are detected in CL/P A/WySn embryos compared to phenotypically normal A/WySn embryos, but the mechanism by which this occurs is unknown [63,67]. On a C57BL/6J (B6J) genetic background with the *Clf2* modifier, the *clf1* IAP element is more highly methylated, no IAP transcripts are detectable, and *Wnt9b* expression is normal [66]. As assessed by Combined Bisulfite and Restriction Analysis (COBRA), phenotypically normal A/WySn embryos appear to exhibit variable DNA methylation at the 5′ LTR of the *clf1* IAP element. This suggests this IAP is a metastable epiallele and it was thus redefined as *Wnt9b^IAP^* by Juriloff et al. [63].

Once again, the modifier responsible for the strain-specific methylation of the CL/P-inducing IAP, *Clf2*, has not yet been identified but has been mapped to a 3Mb region on Chromosome 13; this region contains 48 genes and includes a known KRAB-ZFP cluster of more than 30 KRAB-ZFPs [58,66] (Figure 2). Many of the KRAB-ZFP genes in this cluster contain divergent non-synonymous single nucleotide polymorphisms (SNPs) between the B6J and A/WySn strains [28,66]. Though both are on Chromosome 13, this KRAB-ZFP cluster is distinct from that identified in the mapping experiments of the *Mdac* candidate.

### 3.3. Non-Ecotropic ERV Activation Links to Lupus

The mouse strains commonly used as models for human systemic lupus erythematosus (SLE)—New Zealand Black (NZB), New Zealand White (NZW), and 129—have high gene expression and protein levels of non-ecotropic ERV (NEERV) envelope glycoprotein gp70, concomitant with nephritis. However, a causative link between NEERV dysregulation and lupus pathology has not been established, and the mechanism for NEERV dysregulation is unknown. Previously, independent QTL analyses in the NZB/NZW and 129 strains mapped the loci (*Sgp3* in NZB and *Gv-1* in 129) responsible for the gp70 autoantigen expression to large intervals on Chromosome 13 [68,69].

A recent comparative RNA-seq study between B6J and C57BL/6N (B6N) found that the majority of differentially expressed loci between these two sub-strains were NEERVs; the ERV envelope protein and NEERV gene expression were significantly higher in B6N compared to B6J [70]. F1 hybrid mice showed low NEERV gene expression and ERV envelope protein levels that were comparable to B6J mice, indicating the presence of a dominant NEERV repressor in the B6J sub-strain. A QTL analysis revealed a single QTL locus on Chromosome 13 responsible for NEERV dysregulation; an inter-strain comparison and copy number analysis revealed a 1Mb deletion specific to B6N in the mapped interval. This 1Mb region in B6J contains two genes, four non-coding RNAs, and four pseudogenes (Figure 2). The knockouts of the two genes in this interval on a B6J genetic background phenocopied the NEERV dysregulation seen in the B6N strain. The two genes are the KRAB-ZFP genes *2410141K09Rik* and *Gm10324*, renamed *suppressor of NEERV* (*Snerv*) *1* and *2*.

The previously mapped intervals for *Sgp3* and *Gv-1* in NZB and 129, respectively, include *Snerv1* and *Snerv2*. Additionally, the 1Mb deletion in B6N also appears to be deleted in NZB and 129, indicating that the previously mapped *Sgp3* and *Gv-1* loci may be the same modifiers as *Snerv1* and *2*. Complementation experiments found that hybrid F1 mice (*Snerv1/2^-/-^* X NZB/129) were unable to rescue the NEERV repression phenotype, indicating that the strain-specific absence of these KRAB-ZFPs, previously identified as *Sgp3* in NZB and *Gv-1* in 129, may drive the NEERV dysregulation and lupus pathology in lupus-prone strains, NZB and 129.

### 3.4. IAP-Driven Stabilin2 Expression in DBA/2J Mice

*Stabilin2* (*Stab2*) encodes a type I transmembrane receptor which functions via clathrin-mediated endocytosis as a scavenger receptor for hyaluronans (HA), heparin, and pro-collagen peptides, amongst other macromolecules [71,72]. The main phenotype of *Stab2* null mice, which were generated on B6J and BALB/cJ genetic backgrounds, is a 10-fold higher plasma HA concentration compared to wild type [73,74]. Independently, one study reported that wild-type DBA/2J (DBA) mice have a more than 10-fold higher plasma HA concentration than 129S6 or B6J mice, a phenotype which was mapped to the *Stab2* locus [75]. Recently, it was shown that a 5.5kb IΔ1-type IAP element inserted 220bp upstream of the canonical *Stab2* transcription start site (TSS), providing an alternative TSS which drives the ectopic expression of *Stab2* [76] (Figure 1c, upper panel). The IAP element (*Stab2-IAP*), alternative TSS, and ectopic *Stab2* expression are unique to the DBA genetic background.

In B6J x DBA (BxD) F1 hybrid heart tissue, IAP-driven *Stab2* expression is completely abrogated, indicative of a single dominant modifier in the B6J strain targeting the *Stab2-IAP* for silencing and preventing aberrant transcription. A congenic line homozygous for the DBA-specific *Stab2-IAP* in an otherwise 129S6/Sv (129S6) genetic background exhibited *Stab2* expression levels that were significantly reduced compared to DBA but that were higher than a pure 129S6 genetic background. The lack of the complete transcriptional repression of the IAP-driven transcripts suggests that an additional locus or loci are responsible for targeting the DBA-specific IAP on the 129S6 genetic background. The 5′ LTR of the *Stab2*-IAP is highly methylated on the DBA and 129S6 genetic background, as assessed by clonal bisulfite sequencing (75.7% vs. 85.0%, respectively), indicating that a mechanism besides DNA methylation may also be involved in the strain-specific behaviour of this IAP element [76] (Figure 1C, lower panel). The methylation status of the *Stab2*-IAP was not assessed on a B6J background. The small increase in DNA methylation at the *Stab2-IAP* on a 129S6 genetic background may be a secondary consequence of other repressive epigenetic modifications, such as increased H3K9me3, which prevent ectopic transcription initiating from the LTR. Besides DNA methylation, additional epigenetic modifications at the *Stab2-IAP* were not assayed.

Utilising the BxD recombinant inbred lines and gene expression data from the Hybrid Diversity Panel, the most prominent *trans* expression QTL (eQTL) locus was mapped to Chromosome 13 and refined to 59.7–73Mb [76] (Figure 2). The region overlaps the modifiers (and KRAB-ZFP clusters) mapped for *Mdac* (56–65 Mb), *Clf2* (64.95 -67.9 Mb) and *Snerv1/2* (65.66–66.7 Mb). In the other three examples of strain-specific regulation discussed so far, the modifiers appear to be single dominant loci. This seems to hold true for the *Stab2*-*IAP* in a B6J F1 background. However, on a 129S6 F1 background, the *Stab2-IAP* is not fully repressed, as assessed by the elevated *Stab2* expression and the methylation status of the 5′ LTR. In this regard, the strain-specific modifier acting on *Stab2-IAP* is particularly interesting, as it exhibits both strain-specific absence/presence polymorphism (B6J and 129S6 vs. DBA) and a strain-specific mode of action (B6J vs. 129S6). It is worth pointing out that when a single locus is mapped, there may be multiple modifiers that are always inherited together capable of recognising the target ERV, making it seem like the Mendelian segregation of a single gene. This is especially pertinent given that all of the mapped intervals contain KRAB-ZFP clusters, which are known to expand through segmental duplication, resulting in individual KRAB-ZFPs with highly redundant roles [59].

### 3.5. Epigenetic Inheritance of Metastable Epialleles, A^vy^ and Axin^Fu^

Metastable epialleles are regions of the genome which display variable epigenetic states between genetically identical individuals [77,78]. The most comprehensively studied metastable epialleles to date, *A^vy^* and *Axin^Fu^*, result from the variable silencing of IAP element insertions upstream of, or within, endogenous gene loci, and were identified due to observable phenotypic differences between littermates [79,80,81,82,83]. Whilst no strain-specific modifiers have been identified that specifically act on the IAP elements responsible for the *A^vy^* or *Axin^Fu^* alleles, it is clear that genetic background influences the heritability of these loci as well as the susceptibility of these alleles to environmental stimuli, two features for which these loci are particularly well known.

**Figure 3:**
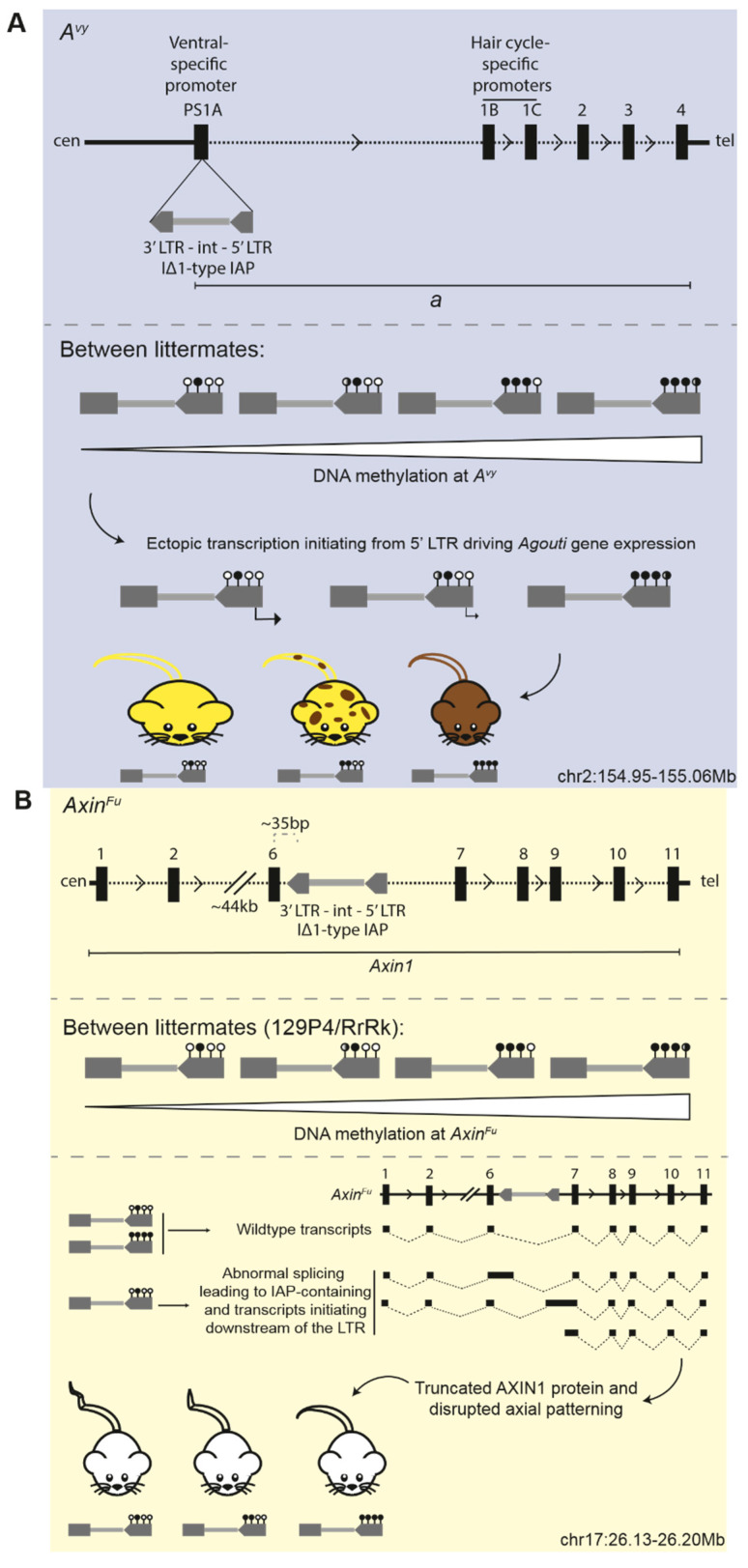
Schematics depicting the insertion sites of the metastable epialleles (**A**) *A^vy^* and (**B**) *Axin^Fu^*. Upper panel shows the intracisternal A-type particle (IAP) insertion relative to the affected gene; lower panel shows the functional consequence of the variably methylated IAP element. Coordinates are mm10. Sticks with closed circles represent methylated CpGs in the LTR of the ERV; sticks with open circles represent unmethylated CpGs. Black dotted lines depict introns; thick black lines depict intergenic regions.

The *A^vy^*-causing IAP insertion first arose spontaneously on a C3H/HeJ (C3H) genetic background in pseudoexon 1A of the coat colour gene *Agouti*, 100kb upstream of the coding exons [79,84] (Figure 3A, upper panel). In wild-type mice, the *Agouti* expression is regulated by a hair cycle-specific promoter; transient expression at the beginning of each hair follicle cycle results in a sub-apical yellow band on an otherwise black hair follicle and the “agouti” coat pattern. In *A^vy^* mice, transcription originating from a cryptic promoter within the LTR of the IΔ1-type IAP element drives the ectopic expression of *Agouti* (Figure 3A, lower panel). The excess paracrine signalling molecule causes hair follicle melanocytes to constitutively synthesise yellow pigment (phaeomelanin), resulting in mice with yellow coats as well as adult-onset obesity, diabetes, and an increased susceptibility to tumours [85,86]. Isogenic individuals have variable DNA methylation at the IAP element, which inversely correlates with ectopic *Agouti* expression levels: highly-methylated individuals retain endogenous levels of expression and are indistinguishable from wild type (termed pseudoagouti); lowly-methylated individuals have high levels of ectopic expression and have a yellow coat, diabetes, and obesity; intermediately methylated individuals have an intermediate mottled coat-colour phenotype [80,84] (Figure 3A, lower panel).

Similarly, the *Axin^Fu^* allele resulted from a spontaneous IΔ1-type IAP element insertion in the sixth intron of *Axin1* in the Bussey Institution stock of mixed genetic backgrounds [81,82] (Figure 3B, upper panel). At a low rate, the intronic IAP causes aberrant splicing, which results in both wild-type and mutant transcripts which contain the IAP; the inclusion of the *Axin^Fu^* IAP in the mRNA is predicted to generate a truncated AXIN1 protein [82] (Figure 3B, lower panel). Transcripts initiating within 100 bp downstream of the 3′ LTR of the *Axin^Fu^* IAP have also been detected and may result in truncated peptides consisting of intron 6 and exons 7–10 [83]. The resultant kinked tail phenotype is attributed to the abnormal development of the posterior somites and axial duplications, which leads to vertebral fusions via atypical Wnt signalling [82,83]. Among isogenic individuals, tails range from kinked to completely normal, with the phenotypic severity inversely correlating with the DNA methylation status of the intronic *Axin^Fu^* IAP element [83] (Figure 3B, middle and lower panels).

**Figure 4:**
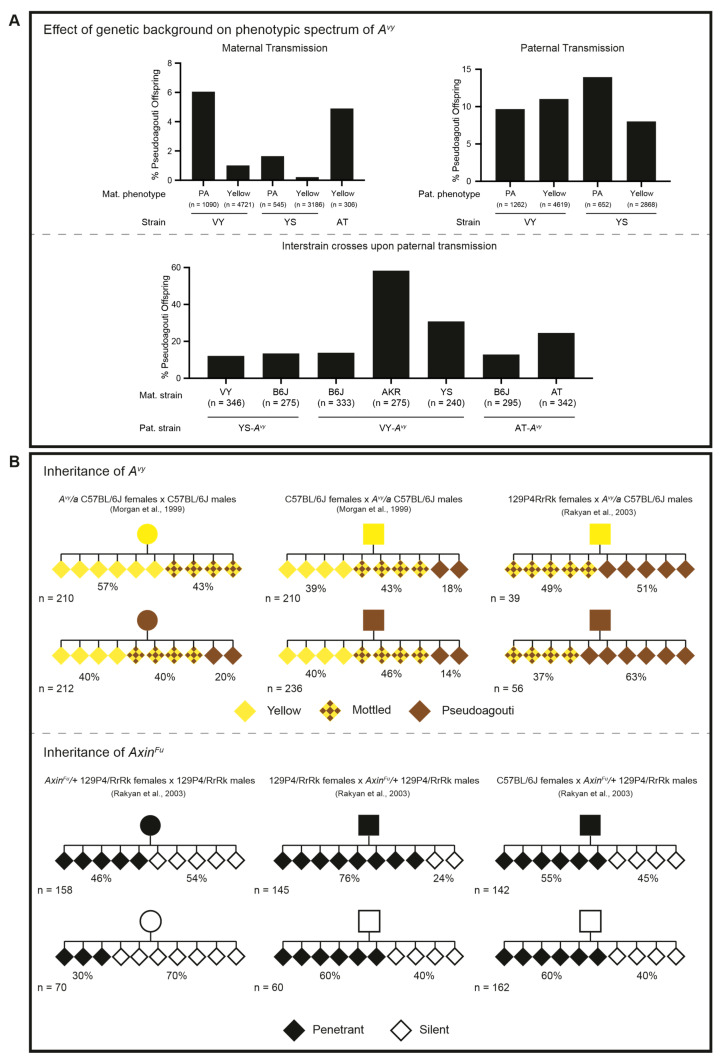
The effect of genetic background on the phenotypic spectrum and inheritance of *A^vy^* upon maternal or paternal transmission. (**A—upper**) Maternal and paternal transmission of the *A^vy^* allele on VY/Wf (VY), YS/ChWf (YS), or AT/Wf (AT) genetic backgrounds. The percentage of pseudoagouti (PA) offspring depends upon the maternal coat colour phenotype, but not the paternal coat colour phenotype. Paternal transmission of *A^vy^* results in a higher percentage of PA offspring than the maternal transmission of *A^vy^*. Both maternally and paternally transmitted alleles are sensitive to genetic background effects, albeit in different ways. (**A—lower**) Paternal transmission of *A^vy^* is largely influenced by the genetic background of the dam. Paternal coat colour information is not included, as it is not available for all of the inter-strain crosses. B6J = C57BL/6J; AKR = AKR/LwNIcr. The data shown in the upper and lower panel are combined (based on the genotype and parent-of-origin) and adapted from [87,89,90]. (**B—upper**) On a C57BL/6J background, the maternal coat colour phenotype influences the coat colour of the *A^vy^*/*a* offspring, but the paternal coat colour phenotype has no effect on the phenotypic distribution of the offspring. When the *A^vy^* allele is paternally inherited through a 129P4/RrRk-fertilised oocyte, the paternal coat colour phenotype influences the coat colour of the *A^vy^*/*a* offspring; the percentage of PA and mottled is increased after passage through a 129P4/RrRk-fertilised oocyte compared to a C57BL/6J-fertilised oocyte. (**B—lower**) On a 129P4/RrRk background, the maternal and paternal tail kink phenotype influences the tail kink phenotype in the *Axin^Fu^/+* offspring. The phenotypic distributions are different upon the maternal versus paternal transmission of the allele. When the *Axin^Fu^* allele is paternally inherited through a C57BL/6J-fertilised oocyte, the tail kink phenotype of the sire has no bearing on the phenotypic distribution in the *Axin^Fu^/+* offspring. The data shown in the upper and lower panel are adapted from [80,83]. Pedigrees: circle—female; square—male; diamond—unspecified.

Hybrid experiments to assess whether the *A^vy^* mutation was pleiotropic provided the first evidence that the *A^vy^* IAP was sensitive to genetic background; 12% of the B6JxVY-*A^vy^* hybrids were pseudoagouti, compared to 58% of the AKR/LwNIcr (AKR)xVY-*A^vy^* hybrids [87] (Figure 4A, lower panel). This may be indicative of an AKR-specific modifier(s) that more robustly recognises the *A^vy^* IAP for silencing and “pushes” the offspring towards the pseudoagouti end of the phenotypic spectrum. Subsequent studies showed that the phenotypic distribution and physiological outcomes of *A^vy^* mice shifted dependent on genetic background [86,88,89,90,91]; similar findings have been reported for the *Axin^Fu^* locus [92,93]. In addition, the phenotypic shifts in the offspring of *A^vy^* dams subjected to methyl-supplemented diets differ depending on the genetic background of the dam, likely reflecting differences in methyl metabolism between strains [90,91]. Figure 4A summarises the breeding experiments on *A^vy^* conducted by Wolff spanning almost 30 years.

Both the *A^vy^* and *Axin^Fu^* alleles display strain-specific epigenetic inheritance across generations (Figure 4B). On a B6J genetic background, *A^vy^* displays partial inheritance when transmitted maternally, whilst the *Axin^Fu^* allele on a 129P4/RfRk (129P4) genetic background is inherited upon both maternal and paternal transmission [80,83]. Interestingly, *A^vy^* also displays paternal inheritance when *A^vy^/a* B6J males are crossed with *Axin^Fu^/+* 129P4 females (Figure 4B, upper panel). Conversely, *Axin^Fu^* is no longer paternally transmitted when *Axin^Fu^/+* 129P4 males are crossed with *A^vy^/a* B6J females, indicating that the inheritance of the *Axin^Fu^* allele is strain-specific, rather than an intrinsic property of the locus [83] (Figure 4B, lower panel). These findings suggest that paternally inherited alleles are subject to the full erasure of DNA methylation during epigenetic reprogramming by B6J-fertilised oocytes, but not 129P4-fertilised oocytes. Indeed, immediately following fertilisation and in line with the rest of the genome, the paternal *A^vy^* allele undergoes rapid demethylation, whereas the maternal *A^vy^* allele does not in the B6J strain [94,95,96]. However, importantly, both the paternal and maternal alleles exhibit a comparable absence of DNA methylation at *A^vy^* at the blastocyst stage, which suggests that DNA methylation is not the inherited epigenetic mark [96].

Our group recently performed a genome-wide systematic screen of IAP elements in B6J to identify novel metastable epialleles, which we termed variably methylated IAP elements (VM-IAPs) [97]. Although VM-IAPs are not commonly associated with transcriptional changes, nor do they retain a memory of the parental methylation level in the offspring, we do find that VM-IAPs are sensitive to genetic background and parent-of-origin effects, in line with the *A^vy^* and *Axin^Fu^* alleles [98]. Given that VM-IAPs are naturally occurring alleles that are polymorphic between strains, they represent an attractive model in which to study the modifiers and mechanisms involved in the strain-specific epigenetic regulation of IAP elements. Taken together, in depth cross-strain analyses on *A^vy^*, *Axin^Fu^*, and VM-IAPs are likely to provide mechanistic insight into the establishment and maintenance of these unique loci.

## 4. Lessons from Transgenes

The similarities between metastable epialleles and the epigenetic targeting of transgenes have been highlighted previously [77,78,99,100,101,102]. Methylated transgenes and endogenous metastable epialleles are loci with varying degrees of DNA methylation that can exhibit parent-of-origin effects upon transmission. In addition, the genetic background in which transgenes and metastable epialleles are studied affects their methylation state and heritability, suggesting that strain-specific modifiers are acting on these loci.

The strain-specific silencing of ERVs is particularly reminiscent of the decades-spanning work on a transgene designed to study the rearrangement of immunoglobulin genes in the Storb lab. The transgene was designed as a marker of V-J recombination: the construct contains a mouse metallothionein-1 promoter upstream of the bacterial *xanthine/guanine phosphoribosyltransferase* (*gpt*) gene, whose translation is dependent on V-J rearrangement to form an upstream in-frame initiation codon [103]. The transgene was named HRD (heavy chain enhancer, rearrangement by deletion), and was found to recombine 100% of the time in a transfected pre-B cell line [103,104]. When maintained on the DBA genetic background, the HRD transgene is unmethylated. Upon crossing the founder transgenic mice to B6J, the HRD transgene becomes highly methylated and no longer undergoes V-J recombination in the lymphoid organs of the offspring [105]. The further crossing of B6J mice with the HRD transgene to DBA or SJL/J (SJL) mice resulted in offspring with a methylated HRD transgene. Crossing these F1 hybrids (B6Jx DBA or SJL) back to DBA or SJL mice resulted in offspring (N1) with an unmethylated, partially methylated, or methylated HRD transgene, suggestive of a dominant B6J modifier that had been lost in some of the backcrossed N1 individuals. The single dominant B6J allele responsible for HRD transgene methylation, *Strain-specific modifier 1 in C57BL/6J* (*Ssm1b*), was found to be concordant with the B allele of *Fv-1*, a resistance allele to the Friend leukaemia virus, which mapped *Ssm1b* to Chromosome 4 [105]. *Ssm1b* was later fine-mapped to a 0.5Mb interval on Chromosome 4, a region containing ~12 genes, six of which are KRAB-ZFPs [106]. Several overlapping bacterial artificial chromosomes (BACs) covering the mapped region were injected into fertilised eggs carrying the HRD transgene on an unmethylated genetic background (C3H x DBA hybrids). Two of the BACs containing a single gene in common resulted in the methylation of the HRD transgene, enabling the identification of *Ssm1b* as *Zfp979* (NCBI designation is *2610305D13Rik*) [106].

*Zfp979* has a KRAB-A box and three functional C_2_H_2_ zinc fingers interspersed with three non-functional zinc fingers. It resides in a cluster which contains 20 other KRAB-ZFPs on Chromosome 4. *Zfp979* is widely expressed during embryonic development until embryonic day 8.5 (E8.5). Whilst ESCs on a DBA background have 0% DNA methylation at the HRD transgene, B6J ESCs have ~40% DNA methylation, indicating that the methylation of the HRD transgene likely occurs peri-implantation, coincidental with the rest of the genome [107]. In a B6J or hybrid BxD background, methylation at the HRD transgene increases from 40% to almost 100% between E8.5 and E9.5 and relies on the *de novo* methyltransferase DNMT3B [106]. The direct binding of ZFP979 to the HRD transgene and the existence of a ZFP979:KAP1 interaction have not yet been shown. A recent analysis of KRAB-ZFP clusters established that ZFP979/2610305D13Rik binds to IAPEY-int elements [59].

*Ssm1b* was originally identified due to the differential methylation of the HRD transgene in B6J (methylated) and DBA (unmethylated) genetic backgrounds. In total, when maintained on an F1 background with DBA or SJL, the HRD transgene is methylated in seven strains of mice (C57BL/6J, FVB/NJ, C57L/J, LP/J, 129/SvJ, BALB/cJ, and A/J) and unmethylated in six strains of mice (DBA/2J, C3H/HeJ, SJL/J, CBA/J, SM/J, and AKR/J) [108]. The reference genomes for 16 strains were recently generated but there remain significant gaps in this region, likely due to the repetitive nature of the KRAB-ZFPs within this cluster, which are predicted to have arisen through segmental gene duplications [17,59,109]. This makes inter-strain sequence alignments of *Zfp979* currently impossible. The generation of new reference genomes using long-read sequencing technologies will alleviate these issues and enable inter-strain comparisons at KRAB-ZFP clusters and other repeat-dense regions in the future.

Indeed, the variable epigenetic state of both transgenes and metastable epialleles has been utilised to screen for modifiers involved in their epigenetic targeting: large-scale N-ethyl-N-nitrosourea (ENU) mutagenesis screens have identified dominant and recessive genes capable of modifying GFP transgene variability in mice [102,110,111,112,113,114,115,116]. The hits from these screens have been named *Modifiers of Murine Metastable Epialleles* (*Mommes*), and the effect of some of these mutants on the phenotypic spectrum of *A^vy^* has been assessed [111,112]. Many of the candidates from the screen are known components of the ERV epigenetic silencing pathway, but it is not yet known if, and to what extent, these modifiers act in a strain-specific manner. In this regard, a screen focussing on the strain-specific modifiers of ERV silencing would be timely.

## 5. KRAB-ZFPs as Effectors of Strain-Specific ERV Regulation

Thus far, the most compelling examples of strain-specific ERV regulation involve IΔ1-type IAP elements, with the exception of the dactylaplasia-causing MusD insertion at *Fbxw4.* This is perhaps unsurprising, given the strain-specific expansions of IAP and MusD/ETn elements [9,11,13,15]. Whilst the two IΔ1-type IAP elements whose modifiers map to overlapping regions, *clf1* and *Stab2-IAP*, are permissive in different strains, they are both targeted for silencing on a B6J genetic background, raising the possibility that their respective modifiers, *Clf2* and *Stab2-modifier*, may in fact be the same (Table 1). As these strain-specific modifiers have been discovered due to observable phenotypes rather than specially designed screens, only dominant-acting alleles have been detected so far. Aside from the NEERV effectors, *2410141K09Rik* and *Gm10324*, specific modifiers for the other ERVs subject to strain-specific regulation are yet to be identified. It is worth noting that many of the modifiers linked to strain-specific ERV regulation reside on Chromosome 13 and overlap known KRAB-ZFP clusters, one of which includes KRAB-ZFPs *Rsl1* and *Rsl2* (Figure 2 and Table 1) [66,70,76,117]. *Rsl1* and *Rsl2* are strain-specific KRAB-ZFPs that regulate sexually dimorphic gene expression in the liver. One of the target genes of *Rsl1*, *sex-limited protein (Slp)*, lies 2kb downstream of an ancient ERV [118,119]. In KAP1 knockout livers, there is an upregulation of *Rsl1*/*Rsl2*-target cytochrome P450 genes, implicating the KRAB-ZFP/KAP1 pathway, with its already established role in ERV silencing, in the functional mechanism of RSL1 and RSL2 [27,120]. The KRAB-ZFP cluster (Chr13.2-cl) containing *Rsl1* and *Rsl2* has previously been identified as being highly variable between mouse strains [121]. It is possible that certain clusters contain KRAB-ZFPs with particularly rapid evolution in response to the amplification of active ERV elements, making them more polymorphic across mouse strains than other clusters. This may explain the strain-specific modifiers mapping predominantly to Chr13.2-cl, as well as the *Ssm1b* cluster, Chr4-cl.

KRAB-ZFPs are attractive candidates for strain-specific modifiers of ERVs due to the sequence specificity endowed by their C-terminal ZFPs, which are under strong positive selection [123,124,125]. Indeed, it is worth noting that many of the ERVs subject to strain-specific regulation are the same type of TE (IΔ1-type IAP: *clf1*, *Stab2-IAP*, *A^vy^*, and *Axin^Fu^*), providing support for the role of sequence in the strain-specific epigenetic targeting of these elements. Furthermore, the positive correlation that exists between the number of LTR retrotransposons and the number of zinc finger domains across mammalian species is indicative of concurrent waves of KRAB-ZFP and TE expansion [124,125]. The varying KRAB-ZFP gene content between strains, and even sub-strains of mice with less than 75 years divergence in the case of B6N and B6J, may underlie differing ERV activity and epigenetic regulation between strains of inbred laboratory mice [17,70,126,127]. In particular, the C3H strain is particularly susceptible to IAP mobilisation, and it is tempting to speculate that this may be explained by a KRAB-ZFP cluster or gene deletion, as has occurred in the B6N, 129, and NZB strains, causing NEERV dysregulation [9,70]. The coexistence of strain-specific ERVs and strain-specific KRAB-ZFPs provides intriguing complexity to the KRAB-ZFP/TE coevolution debate in light of the non-mutually exclusive “arms race” and “domestication” models [27,28,128]. Future attempts to functionally characterise strain-specific KRAB-ZFPs in detail may be difficult. In addition to the high prevalence of gaps currently in the reference genomes at KRAB-ZFP clusters and repetitive regions, the high level of redundancy in the KRAB-ZFP-targeting of ERVs will make the identification and validation of candidate modifiers challenging [23,125,129]. This high level of redundancy may explain why KRAB-ZFPs were not identified in the *Mommes* mutagenesis screen [102,116].

It is not yet clear whether modifier loci are required for the establishment or maintenance of strain-specific epigenetic states at ERVs and transgenes. The identification and emphasis of KRAB-ZFPs as strain-specific modifiers so far in this review suggests the focus may be on the strain-specific “establishment” of an epigenetic state, although some KRAB-ZFPs are known to play a protective role maintaining germline-derived DNA methylation marks during early embryonic development, notably ZFP57 and ZFP445 at imprinted loci [32,130,131,132]. Whilst 75% of ZFP57 binding sites are located in ERVs, the loss of this protein does not affect H3K9me3 deposition or DNA methylation at ERVs, nor does it lead to the loss of transcriptional repression of ERVs in ESCs [130,131,133]. This perhaps reflects the highly redundant nature of KRAB-ZFP-mediated ERV repression, or it may suggest that ZFP57 does not play a role at these TEs. Interestingly, instances of strain-specific ZFP57 binding have been reported previously, conferred by genetic variation either in the ZFP57 binding motif itself or in neighbouring regions between strains, causing strain-specific differential methylation and subsequent ZFP57 binding [131]. Recently, the strain-specific loss of imprints in ESCs (129 v B6J) was mapped by QTL analysis to an interval spanning 52Mb-67.7Mb on Chromosome 13, overlapping entirely or partially with the *Mdac*, *Clf2*, *Snerv1/2*, and *Stab2*-modifier loci [134]. Needless to say, the establishment of a strain-specific epigenetic state may occur via mechanisms outside of the KRAB-ZFP targeting pathway. Whilst our understanding of these processes is limited, it is possible that strain-specific epigenetic states could arise through the strain-specific protection (by KRAB-ZFPs or other proteins) or the strain-specific removal of epigenetic modifications during epigenetic reprogramming or at other time points in development.

## 6. Concluding Remarks

It is well established that the genetic background of mouse models can result in large phenotypic differences not attributable directly to the phenotype-associated genetic locus itself. Historically, these strain differences have been largely overlooked due to the technical challenges associated with identifying the underlying modifier genes. This has resulted in specific strains of mice being used in the study of certain traits, as is the case with the “A” strain mice and clefting. We note that while attention to genetic background as a variable across experiments is necessary to ensure experimental reproducibility as well as cross-strain, and potentially cross-species, generalisability, we hope that here we have emphasised the biological and mechanistic value of cross-strain experiments. Currently, the annotations of KRAB-ZFP clusters are poor even in the C57BL/6J reference genome. Advances in long-read sequencing technologies and the resultant high-quality mouse strain reference genomes with full coverage over KRAB-ZFP clusters and their target TEs will be required to enable large-scale inter-strain experiments and provide further mechanistic insight into the complex relationship between repetitive elements, the KRAB-ZFP machinery, and their coevolution.

The strain-specific modifiers outlined in this review were identified due to observable phenotypic differences between permissive and repressive strains. The extent to which polymorphic ERVs, and indeed polymorphic ERV regulation, act as drivers of phenotypic variation between inbred mouse strains remains unclear. However, it is important to note that inbred laboratory mouse strains suffer from severe inbreeding depression, which may put an unusual strain on the host defence mechanisms against TE mobilisation. Experiments using wild-derived mice alongside laboratory strains would help elucidate whether these effects are reflective of host-TE dynamics in natural populations. Additionally, the studies discussed in this review serve as an important reminder that seemingly complicated epigenetic phenomena are sometimes explained by underlying genetic differences, highlighting the mutual dependence and interrelatedness of genetic and epigenetic pathways.

## Figures and Tables

**Table 1:** Overview of mapped strain-specific modifiers.

Disrupted Locus (ERV Type)	Documented Strains with ERV Mutation	Strain-Specific Phenotype	Dominant Modifier	Documented Permissive Strains	Documented Repressive Strains	Coordinates of Modifier Locus (mm10)	References
*Dac^1j/2j^* (MusD)	*Dac^1j^*—SM/Ckc *Dac^2j^*—MRL/MpJ	Dactylaplasia	*Mdac*	BALB/cJ, A/J, 129/J, SM/Ckc, LG/Ckc,	CBA/J, C3H/J, C57BL/6J, DBA/2J, AKR/J, SWR/J	chr13:56–65Mb	[54,55,56]
*clf1* (IΔ1-type IAP)	A/HeJ, A/WySnJ, A/J	Cleft lip with palate	*Clf2*	“A” strains	C57BL/6J	chr13:64.95–67.9Mb	[63,66,122]
NEERVs	-	NEERV dysregulation and lupus pathology	*Snerv1* and *2*	C57BL/6N, 129S1/Sv, NZB	C57BL/6J	chr13:65.66–66.7Mb	[70]
*Stab2-IAP* (IΔ1-type IAP)	DBA/2J	Elevated plasma HA	*Stab2-modifier*	DBA/2J	C57BL/6J, 129S6/Sv	chr13: 59.7–73Mb	[76]
HRD transgene	-	Transgene no longer undergoes V-J recombination	*Ssm1b*	DBA/2J, C3H/HeJ, SJL/J, CBA/J, SM/J, AKR/J	C57BL/6J, FVB/NJ, C57L/J, LP/J, 129/SvJ, BALB/cJ, A/J	chr4: 147.4–147.9Mb	[108,109,111]
